# Perityphlitic Abscess, Treated with Aspiration through Rectum

**Published:** 1879-12

**Authors:** E. D. Merriam

**Affiliations:** Conneaut, Ohio


### PERITYPHLITIC ABSCESS, TREATED WITH ASPIRATION THROUGH RECTUM.

REPORTED BY E. D. MERRIAM, M. D., CONNEAUT, OHIO.

The BUFFALO MEDICAL AND SURGICAL JOURNAL was particularly welcome when, on the reception of the October number, I perused the very interesting article on Perityphlitic Abscess, by Dr. Herman Mynter. I had just diagnosed a case in my own practice, which I regard of sufficient interest to relate. I was called to see Charles Salisbury, Sept. 25th, 1879; aged 19 years; found pain and tenderness in the right ileo-cæcal region, which, for the first few days, extended considerably over the abdomen, but under hot fomentations and anodynes, the general peritonitis subsided, leaving a circumscribed tumefaction with pain and tenderness in the right iliac region. The fever was not high, nor the pulse above 100 at any time during the whole course of the disease. A blister applied on the eighth day drew well, and by the eleventh day the tumefaction was diminished, and at the same time, dysuria, with some distention in the pelvic region, was making its appearance; the bowels moved without pain; urination frequent, painful and scanty; on the twelfth and thirteenth days there appeared a slight watery and mucous discharge from the rectum, and by digital examination, per rectum, fluctuation was discovered by pressing at the right side of the rectum, about two and a half inches above the anus; on the fourteenth day, assisted by Dr. Fifield, I punctured the abscess through the rectum with the largest needle of Dieulafoy's aspirator, and drew off one and three-fourths pints of fetid pus. the last ounce of which was somewhat mixed with blood, indicating the thorough evacuation of the cavity; from this time the patient made a speedy recovery, and was discharged in two weeks. The point of spécial interest in this case to me was the fact that not a drop of pus was discharged after the first evacuation of the abscess. The immediate closing and healing of so large a cavity was unexpected.

